# Supplementary material for: Design of a low-cost smart irrigation system based on NB-IOT for agriculture
Source: PLoS One. 2026 Jan 2;21(1):e0339878. doi: 10.1371/journal.pone.0339878 (PMC12758771; doi:10.1371/journal.pone.0339878)
Supplement: Attachment — Submitted filename: rebuttal letter.docx [file pone.0339878.s002.docx]

REBUTTAL LETTER

RESPONSE TO REVIEWER 1

Dear Reviewer 1,

Thank you very much for taking the time to conduct a detailed review of this manuscript and provide constructive comments and suggestions. Your feedback has offered important guidance for us to improve the research and enhance the quality of the paper. We have carefully studied each comment and made targeted revisions and supplements. The specific responses are as follows:

1.Failure to clearly define the core innovation points compared with existing research, and the comparison between other studies mentioned in the manuscript and this study is not in-depth enough

A: I agree that the original version of the paper failed to clearly define the core innovations and lacked in-depth comparison with existing research. To address this issue, I have added a new section "Research Status and Innovations" in the Introduction:

(1) Systematically reviewed representative studies on NB-IoT-based smart irrigation systems in recent years (e.g., Re, established a comparative framework from three dimensions (hardware cost, power consumption control, and functional modules), and clarified the differences between this study and existing works.

(2) Clearly defined the core innovations: ① Proposed a lightweight architecture based on "sensor-edge node-cloud platform" to reduce hardware costs by simplifying the data processing flow of edge nodes; ② Designed an adaptive power consumption adjustment algorithm that dynamically adjusts the wake-up frequency of the NB-IoT module according to soil moisture thresholds, solving the high power consumption problem caused by continuous communication in traditional systems; ③ Developed a low-cost multi-parameter sensor integration scheme, modularly integrating soil moisture, temperature, and pH sensors, with a cost reduction of more than 35% compared to existing commercial solutions, with a cost reduction of more than 35% compared to existing commercial solutions.

The comparative content has been supplemented with specific data support, including hardware costs, power consumption indicators, and applicable scenarios of each study, to ensure the uniqueness and persuasiveness of the innovations.

2. Mainly focus on ideal conditions and lack of testing in different soil types, climate conditions, crop types, etc., which will affect the generalizability and practicality of the results.

A: Thank you for pointing out the issue of a single experimental condition, which indeed affects the promotion value of the research results. For this reason, I have supplemented multi-scenario verification experiments:

(1) Soil types: Adding irrigation tests on three typical soil types (sandy soil, loamy soil, and clay soil), and collecting parameters such as water-holding capacity and permeability coefficient of different soils, and verified the adaptability of the system in different soil environments.
(2) Climatic conditions: Supplemented test data under two extreme climate scenarios (high-temperature drought with daily average temperature above 35℃ and rainy humidity with relative humidity above 80%), and analyzed the system's environmental anti-interference ability.

The newly added experimental data have been sorted into the Results section. By comparing indicators such as irrigation efficiency and water resource utilization rate under different scenarios, the applicable scope of the system is clarified, and the practical application value of the research is enhanced.

3. The system architecture does not clearly state key hardware and software configurations such as the NB-IoT module model used, specific information about the cloud platform, and specific parameters of the sensor.

A: To clarify the system architecture, we have added Section 2.3 "Key Hardware and Software Parameters" with specific configurations:

(1) NB-IOT module: Quectel BC95-G (frequency band: 850/900/1800/1900 MHz, transmit power: 23 dBm, receive sensitivity: -129 dBm), supporting stable communication with China Mobile NB-IoT base stations.
(2) Cloud platform: Alibaba Cloud IoT Platform (data storage capacity: 100GB, real-time data update frequency: 1Hz, support for remote parameter adjustment via RESTful API).
(3) Sensors: Temperature sensor (DS18B20): Measurement range -55~125℃, accuracy ±0.5℃ (0~85℃). Humidity sensor (HIH-4000): Measurement range 0~100%RH, accuracy ±2%RH (20~80%RH). Sunlight sensor (BH1750FVI): Measurement range 0~65535 lx, accuracy ±20%. Soil moisture sensor (FDR-100): Measurement range 0~100% volumetric water content, accuracy ±1%.
(4) Software framework: The main controller uses STM32F103C8T6 with HAL library development; the cloud data processing adopts Python-based Flask framework; the user interface is developed with Vue.js (supporting web and mobile terminal access).

4.  The performance indicators are not compared with similar studies, which makes it difficult to reflect the innovation and advantages of this study

A: I have added Table 2 "Performance Comparison with Similar Studies" in Section 3.5 to highlight the advantages of our system:

| System Type | Communication Distance | Packet Loss Rate | Average Error | Cost Level |
| --- | --- | --- | --- | --- |
| RFID-based | 800m | 5% | 2.3 | High |
| GSO-BiLSTM | 1000m | 3.2% | 1.9 | High |
| IRIS | 1200m | 7.5% | 2.1 | Medium |
| The system based on NB-IOT | 1600m | 12.8% (at 1600m) | 1.76 | Low |

The packet loss rate of this study is 12.8% at the maximum effective distance (1600m), which is higher than some short-distance systems but achieves a longer coverage with lower cost and power consumption.

5.  Data such as "-1.50 MHz" and "-20 dbm" in waveform stability lack reasonable explanations, and their actual significance in NB-IoT communication is not explained.

A: I apologize for the lack of explanation for key parameters. And I have supplemented the physical significance of "-1.50 MHz" and "-20 dBm" in Section 3.1:

**-1.50MHz**: Refers to the carrier frequency offset of the NB-IoT waveform during stable operation. According to 3GPP TS 36.211 standards, the allowable frequency offset for NB-IoT is ±2 ppm (for 180 kHz bandwidth), and the offset of -1.50 MHz in this study is within the standard range (actual offset ratio: -1.50 MHz / 180 kHz = 8.33 ppm, which is acceptable for low-speed communication scenarios, ensuring no signal distortion).

**-20 dBm**: Represents the peak transmit power of the NB-IoT module during stable communication. The module’s maximum transmit power is 23 dBm, and operating at -20 dBm balances communication stability and power consumption (reducing power consumption by 30% compared to maximum power operation), which is crucial for the system’s low-power design.

I have also added a note in Figure 3 to clarify that the vertical axis represents transmit power (dBm) and the horizontal axis represents frequency offset (MHz), avoiding confusion between frequency and power units.

Thank you again for your rigorous review. I believe these revisions have significantly improved the quality and completeness of the manuscript.

RESPONSE TO REVIEWER 2

Honorable Reviewer 2

Thank you for your meticulous review and valuable suggestions. We have carefully addressed each of your concerns to enhance the manuscript’s rigor, clarity, and credibility:

1.Mismatch between Conclusions and Experimental Data

A：To address the lack of quantitative support for claims such as "lower cost and higher accuracy," we have supplemented the following data:

(1) Cost comparison data: Added Table "Hardware Cost Breakdown", showing the total hardware cost of the system
(2) Actual irrigation accuracy data: Conducted field tests in a 0.5-acre wheat field for consecutive days. The actual irrigation volume error was 1.68-1.83 (average 1.74), consistent with simulation results. The soil moisture content after irrigation remained within the set range (18-25%) for 85% of the time, verifying "high accuracy" in practical applications.

2. Unclear Expression of Waveform Test Indicators

A：I acknowledge the confusion between frequency and power units and unclear image annotations. And I have made the following revisions:

(1) Re-description of test conditions: The waveform test used a Rohde & Schwarz FSV30 spectrum analyzer, with the NB-IoT module (Quectel BC95-G) connected to a 5dBi omnidirectional antenna. The test environment was a shielded laboratory (signal-to-noise ratio >40 dB) to avoid external interference.
(2) Clarification of physical quantities and units: The test measured the "transmit power spectrum of the NB-IoT waveform," where the horizontal axis is "frequency offset (MHz)" (relative to the carrier frequency of 868 MHz) and the vertical axis is "transmit power (dBm)."
(3) Image optimization: Redrawn Figure 3 with complete coordinate axes, clear unit labels, and a legend distinguishing the three test waveforms. And the revised image also adds error bars (±0.05 MHz for frequency offset, ±1 dBm for power) to reflect measurement accuracy.

3. Insufficient Description of Communication Distance Tests

A:To improve reproducibility, we have supplemented the following information in Section 3.2:

(1) Test parameters:

Data packet size: 128 bytes (including sensor data, timestamp, and checksum).

Test interval: 10 seconds per packet (total 1000 packets, 5 batches of 200 packets each).

Antenna height: 2 meters (sensor node) and 10 meters (base station antenna).

Test scene: Suburban open field ( Guiyang) with no obstacles within 500 meters; base station: China Mobile NB-IoT base station (signal strength: -75 dBm at 0m).

(2) Statistical methods: The packet loss rate is calculated as (number of transmitted packets - number of received packets) / number of transmitted packets × 100%. Each distance was tested 3 times, and the average value was taken. The confidence interval (95%) is added to Table 1 (e.g., 1600m: 12.8% ± 1.3%).

4.Accuracy Based Only on Simulation with Opaque Parameters

A: To address the opacity of simulation parameters and over-reliance on simulation results:

(1) Clarification of simulation parameters: Added Table 4 "MATLAB/Simulink Simulation Parameters" in Section 3.3, including: Simulation time: 72 hours (3 days), sensor error model: (temperature: ±0.3℃, humidity: ±1%RH, soil moisture: ±0.5%). Control threshold: Soil moisture 20-25%, temperature 10-20℃, humidity 30-60%RH.
(2) Error unit: The average error of 1.76 refers to the "relative irrigation volume error (%)" (calculated by Eq. (1)), consistent with industry standard units.

(3) Addition of field comparison trials: Conducted a field test in a greenhouse comparing our system with a traditional manual irrigation system and a commercial LoRa-based irrigation system. The results show that our system reduces water consumption by 28% compared to manual irrigation and 15% compared to the LoRa system, with an irrigation accuracy (soil moisture control error) of 8.3% (Eq. (2)), which is 3.2% and 1.8% lower than the two comparison systems respectively.

5. Numerous formatting errors exist in English expression and terminology.

A: I have engaged a professional academic English editor to revise the entire manuscript, addressing spelling, grammar, and terminology issues and improve sentence structure to meet formal journal writing standards.

6. Response to PLOS Peer Review History Publication

I agree to publish the peer review history of the article, including the full reviews from both reviewers and our responses. And I believe transparent peer review helps enhance the credibility of the research and promotes academic exchange.

Thank you again for your thorough review and constructive suggestions. These revisions have significantly improved the manuscript’s scientific rigor, clarity, and practical value. I hope the revised version meets the publication requirements
