# Supplementary material for: Design of a low-cost smart irrigation system based on NB-IOT for agriculture
Source: PLoS One. 2026 Jan 2;21(1):e0339878. doi: 10.1371/journal.pone.0339878 (PMC12758771; doi:10.1371/journal.pone.0339878)
Supplement: Attachment — Submitted filename: Rebuttal letter (2).docx [file pone.0339878.s003.docx]

REBUTTAL LETTER

RESPONSE TO REVIEWER 1

Dear Reviewer 1,

Thank you very much for taking the time to conduct a detailed review of this manuscript and provide constructive comments and suggestions. Your feedback has offered important guidance for us to improve the research and enhance the quality of the paper. We have carefully studied each comment and made targeted revisions and supplements. The specific responses are as follows:

1.It is recommended to supplement the explanation of the acceptability of a 12.8% packet loss rate in agricultural applications, for example, by comparing it with the data integrity requirements of agricultural irrigation systems. An analysis of the impact of different packet loss rates on irrigation accuracy could be added.

A： Thank you for your comment, I have made careful revision.

2. The manuscript mentions "The average irrigation error of the system is 1.76 (simulation) and 1.74 (field test)," but does not clearly state the unit of measurement and specific definition of the error.

A： Thank you for your comment, I have made careful revision.

3. The manuscript mentions sensor accuracy but does not analyze the relationship between sensor accuracy and the overall system accuracy.

A： Thank you for your comment, I have made careful revision.

4. The manuscript emphasizes the system's low-cost advantage but does not fully discuss its scalability in real-world agricultural environments.

A： Thank you for your comment, I have made careful revision.

5. The supplementary multi-scenario testing is a positive improvement, but the description is rather brief.

A： Thank you for your comment, I have made careful revision.

6. Before CONCLUSION, let's add a section: Discussion and Implication, discussing the potential practical applications of the model proposed in this paper.

A： Thank you for your comment, I have made careful revision.

Response To REVIER 2

This article has a clear research objective and a well-defined problem-oriented approach. It possesses a complete system architecture, detailed experimental data, and a comprehensive literature review. The article's conclusions are reflective, pointing out limitations such as sensor dependence and power supply sustainability, and proposing directions for future improvements. This article completes the entire technical chain from architecture design to performance verification, demonstrating strong engineering implementation capabilities and application value.

A: Thank you for your comment. I sincerely appreciate your work.
